# Evaluating Psychiatry Trainee Exposure to Emergency Department and Acute Hospital Referrals Across Liaison Services: An Audit and Quality Improvement Project

**DOI:** 10.1192/bjo.2026.11715

**Published:** 2026-06-30

**Authors:** Ahmed Ghaly

**Affiliations:** Greater Manchester Mental Health NHS Foundation Trust, Manchester, United Kingdom

## Abstract

**Aims:**

Exposure to acute psychiatric referrals from the Emergency Department (ED) and acute hospital wards is a core component of psychiatry training. With the expansion of Liaison Psychiatry services, many assessments are now managed by non-medical practitioners, raising concerns about reduced trainee involvement. This project aimed to assess current psychiatry trainee exposure to ED and acute ward referrals across liaison services, identify barriers to involvement and supervision, and implement service-level changes to improve training opportunities.

**Methods:**

A two-stage mixed-methods audit was conducted across multiple sites within a large NHS mental health trust. In the first quantitative cycle, an anonymised electronic questionnaire was distributed to psychiatry trainees completing or commencing placements, assessing frequency of exposure to ED and acute ward referrals, supervision quality, and confidence in achieving required competencies (n=44). Following identification of low and variable exposure, a qualitative component was undertaken using semi-structured feedback from liaison psychiatry consultants (n=7) to explore perceived barriers and potential service improvements. Findings were triangulated and presented to the Trust’s Postgraduate Medical Education Committee to inform intervention planning.

**Results:**

The majority of trainees reported limited or infrequent exposure to ED and acute hospital psychiatric referrals despite participating in on-call rotas. Although most trainees described some level of support, a significant minority felt under-supervised. Only a modest proportion expressed confidence in achieving competency in managing acute referrals by the end of placement. Qualitative analysis identified key barriers, including workload pressures,ward prioritisation during on-call shifts, rota structures lacking formal liaison integration, nursing-led triage models limiting trainee involvement, role ambiguity, and trainee anxiety related to confidence and safety.

Following review, Trust-level service changes were approved, including a mandatory one-month liaison shadowing period for new trainees and structured trainee involvement in ED and acute ward referrals with supervision.

**Conclusion:**

This audit demonstrated a system-wide reduction in psychiatry trainee exposure to emergency and acute hospital referrals within liaison services, driven by structural, operational, and behavioural factors. The mixed-methods approach enabled the identification of modifiable barriers and informed the implementation of targeted service redesign to enhance training opportunities.

A planned second audit cycle will evaluate the impact of these interventions on trainee exposure, supervision quality, and confidence. This project highlights the importance of aligning service models with training needs to ensure competency development in acute psychiatric care.

